# Supplementary figures and images for: PI3Kα inhibition blocks osteochondroprogenitor specification and the hyper-inflammatory response to prevent heterotopic ossification
Source: eLife. 2025 Jun 17;12:RP91779. doi: 10.7554/eLife.91779 (PMC12173460; doi:10.7554/eLife.91779)

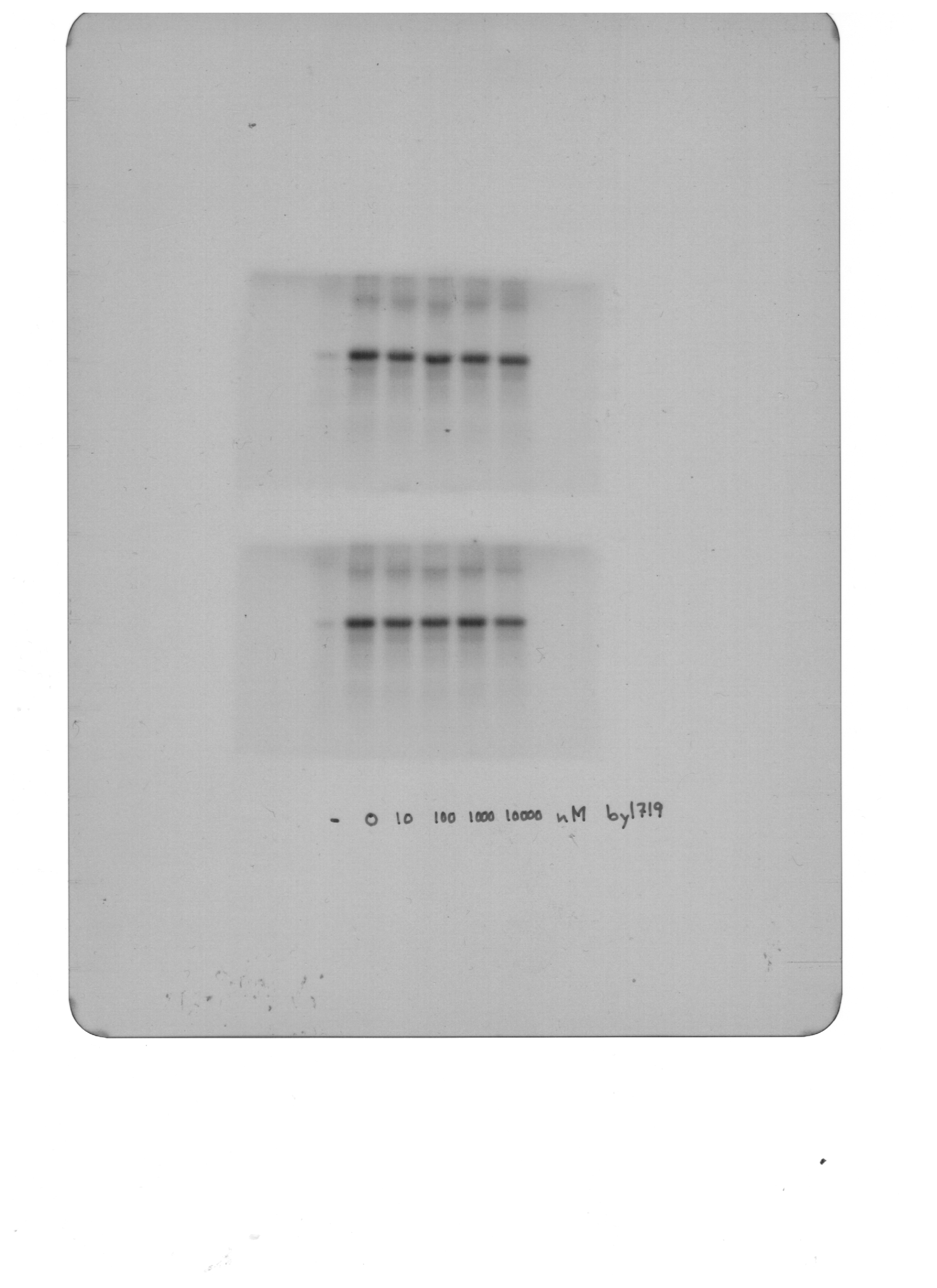

Supplement: Figure 4—source data 1. [file elife-91779-fig4-data1.zip › Figure 4D source data 1/Figure 4D raw data 2.tif]

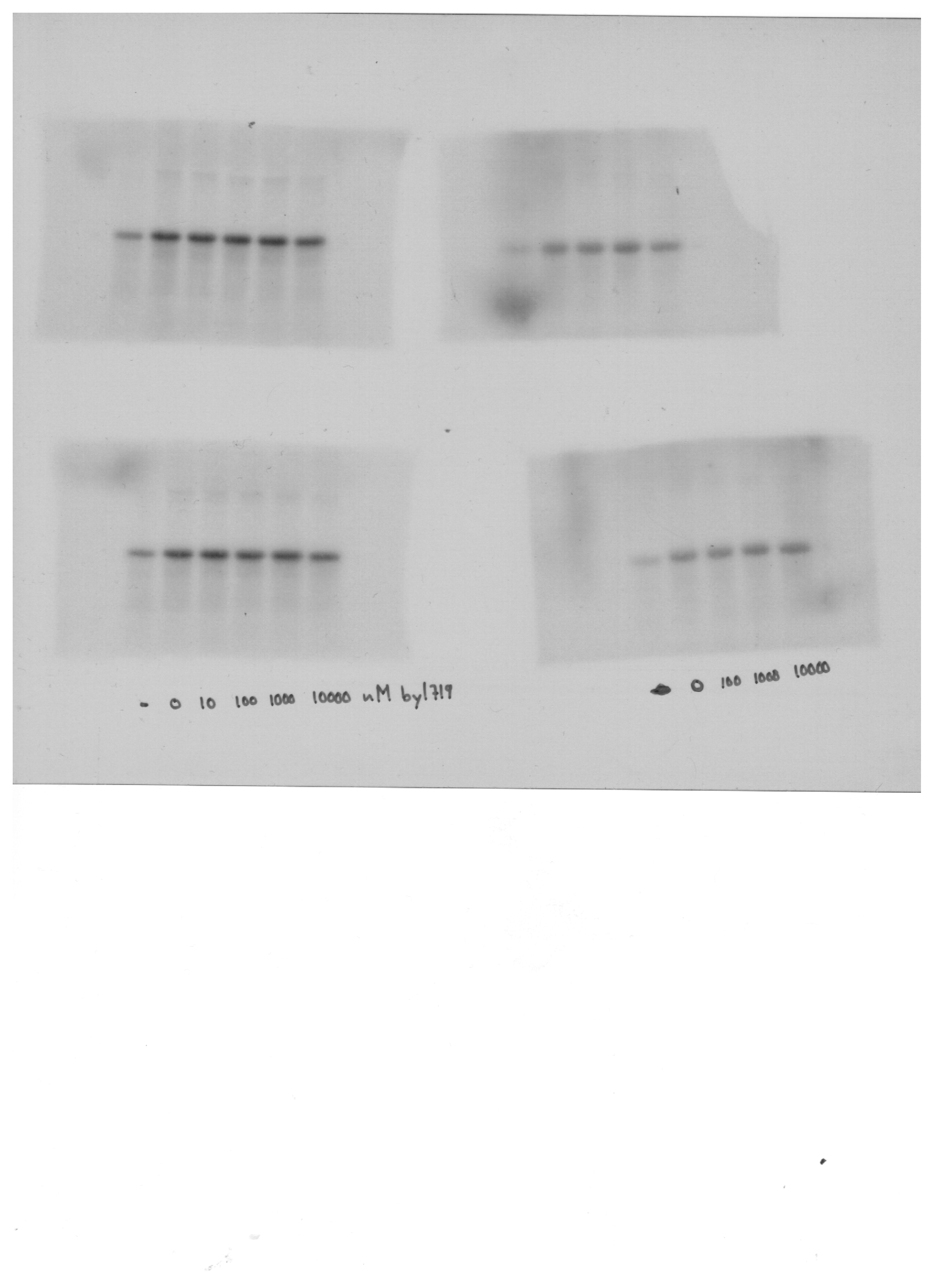

Supplement: Figure 4—source data 1. [file elife-91779-fig4-data1.zip › Figure 4D source data 1/Figure 4D raw data.tif]

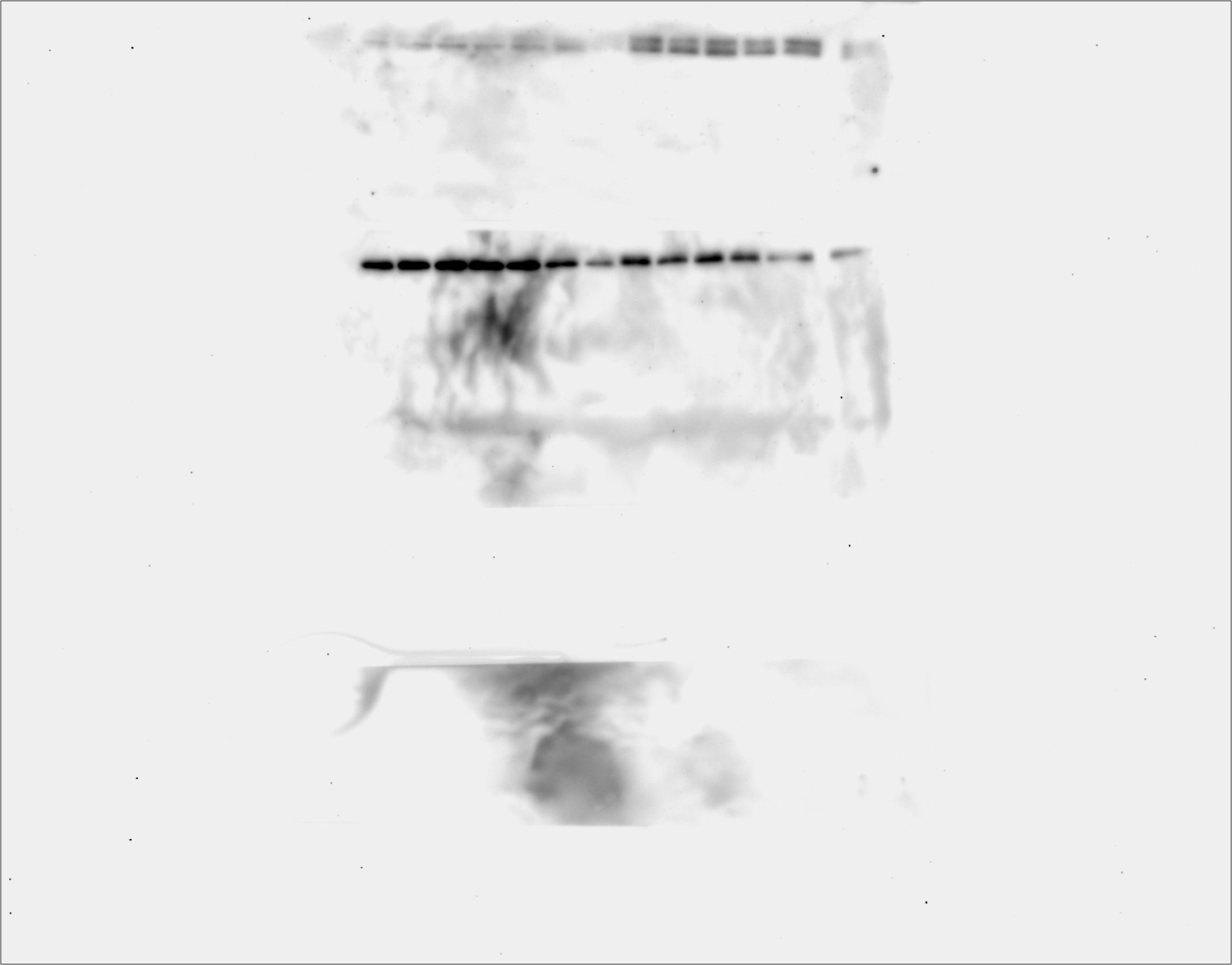

Supplement: Figure 5—figure supplement 1—source data 1. [file elife-91779-fig5-figsupp1-data1.zip › Figure 5 Supplement 1 source data 1/Figure 5 Suppl. 1 pSmad raw data.tif]

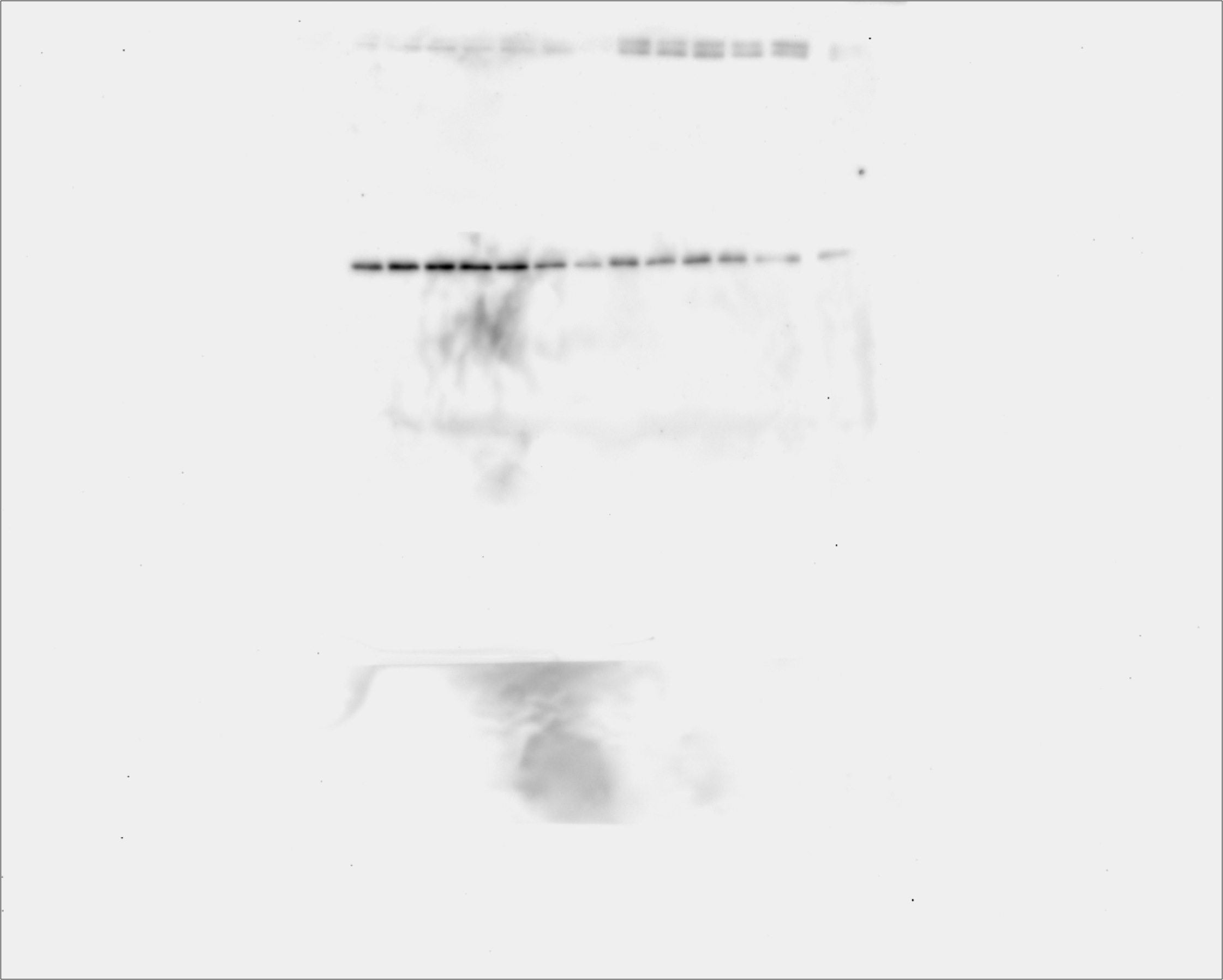

Supplement: Figure 5—figure supplement 1—source data 1. [file elife-91779-fig5-figsupp1-data1.zip › Figure 5 Supplement 1 source data 1/Figure 5 Suppl. 1 SMAD1 total raw data.tif]

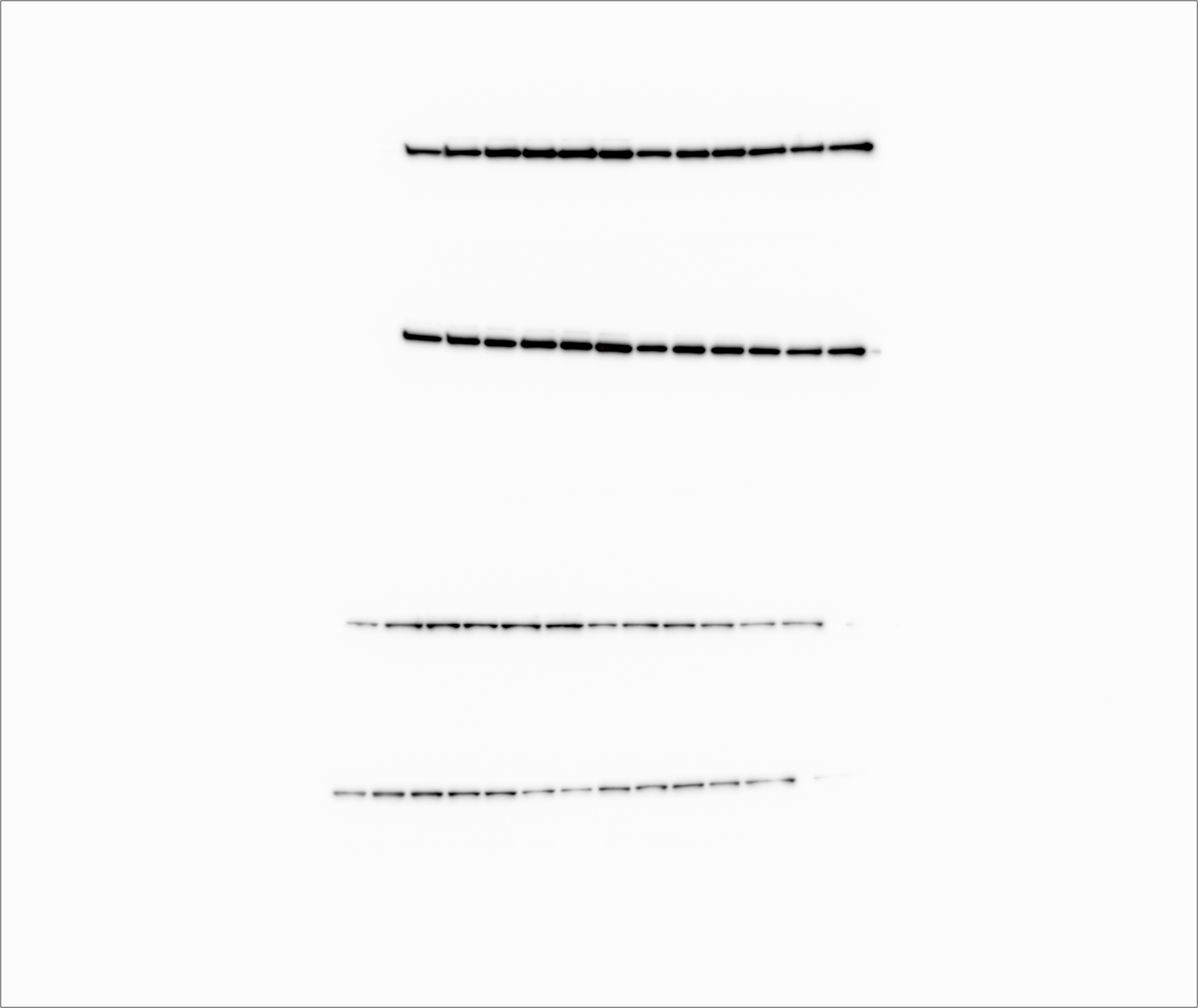

Supplement: Figure 5—figure supplement 1—source data 1. [file elife-91779-fig5-figsupp1-data1.zip › Figure 5 Supplement 1 source data 1/Figure 5 Suppl. 1 vinculin raw data.tif]

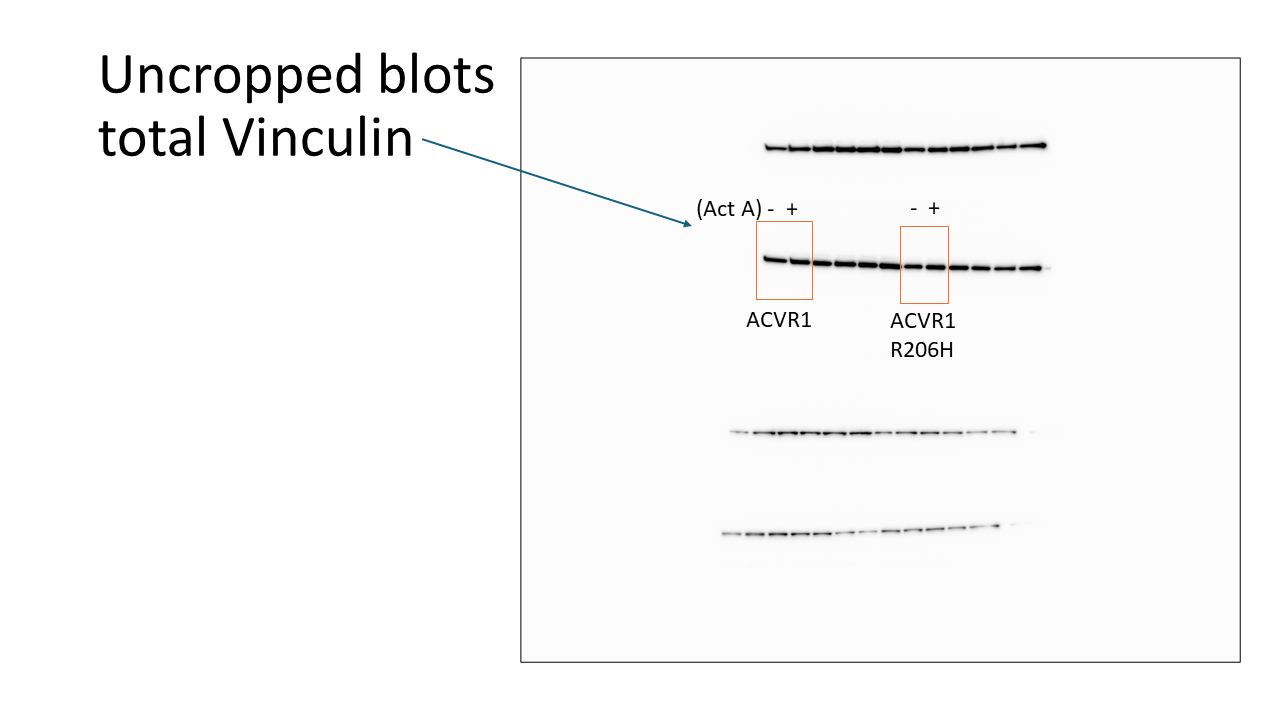

Supplement: Figure 5—figure supplement 1—source data 2. [file elife-91779-fig5-figsupp1-data2.zip › Figure 5 Supplement 1 source data 2/Figure 5 Supplemental 1 labeled uncropped blots Vinculin.tif]

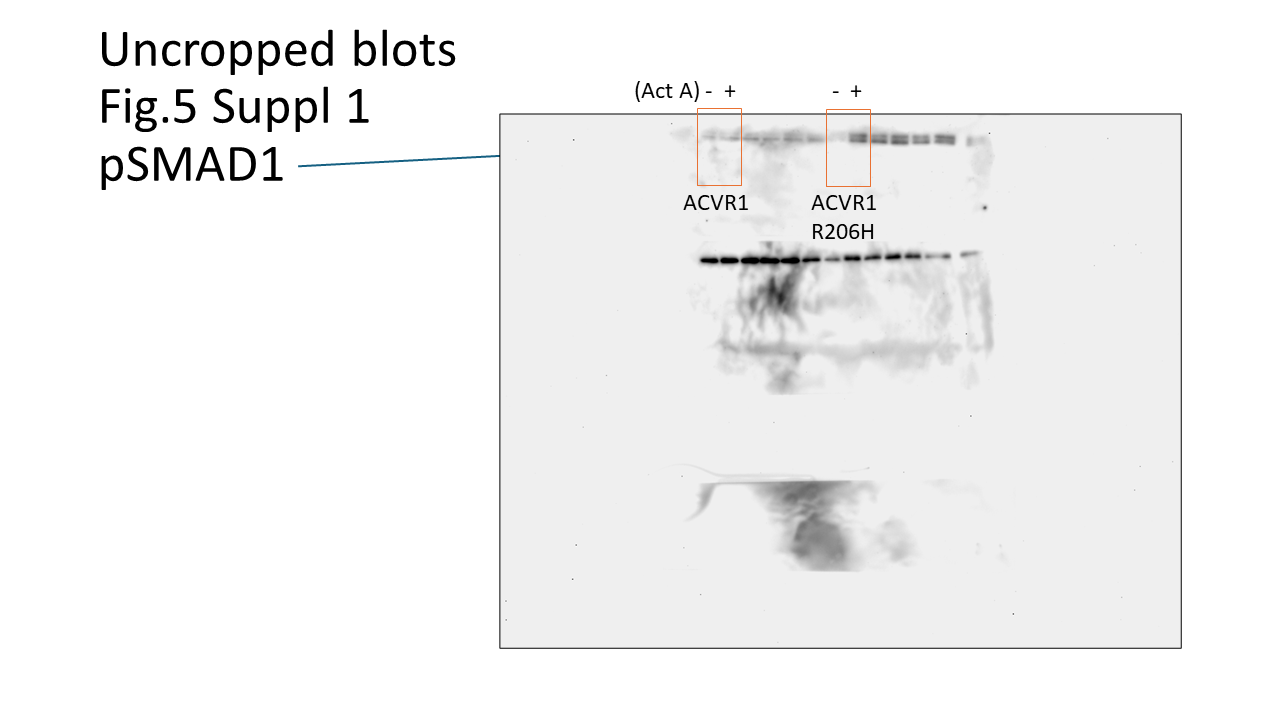

Supplement: Figure 5—figure supplement 1—source data 2. [file elife-91779-fig5-figsupp1-data2.zip › Figure 5 Supplement 1 source data 2/Figure 5 Supplemental 1 labeled uncropped blots.tif]

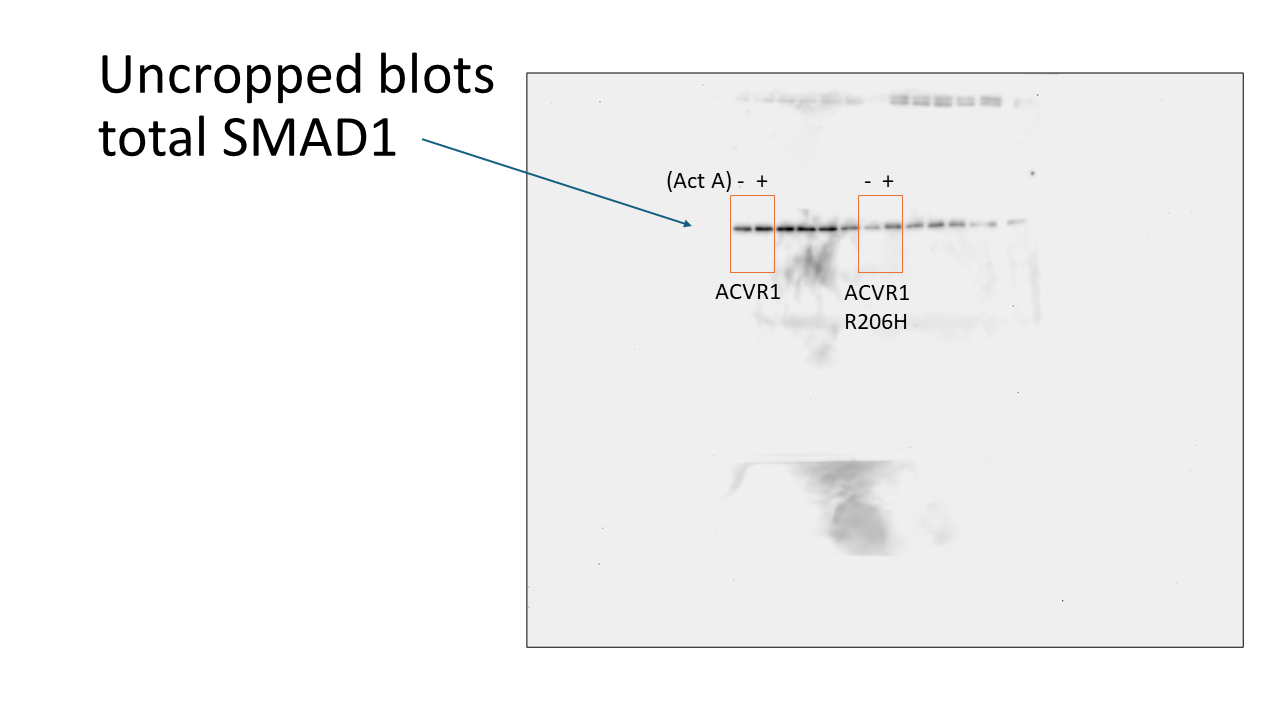

Supplement: Figure 5—figure supplement 1—source data 2. [file elife-91779-fig5-figsupp1-data2.zip › Figure 5 Supplement 1 source data 2/Figure 5 Supplemental 1 labeled uncropped SMAD1 total.tif]
