# Supplementary material for: PI3Kα inhibition blocks osteochondroprogenitor specification and the hyper-inflammatory response to prevent heterotopic ossification
Source: eLife. 2025 Jun 17;12:RP91779. doi: 10.7554/eLife.91779 (PMC12173460; doi:10.7554/eLife.91779)
Supplement: Figure 4—source data 2. [file elife-91779-fig4-data2.zip › Figure 4D source data 2/Figure 4D source data with labelled bands.pdf]

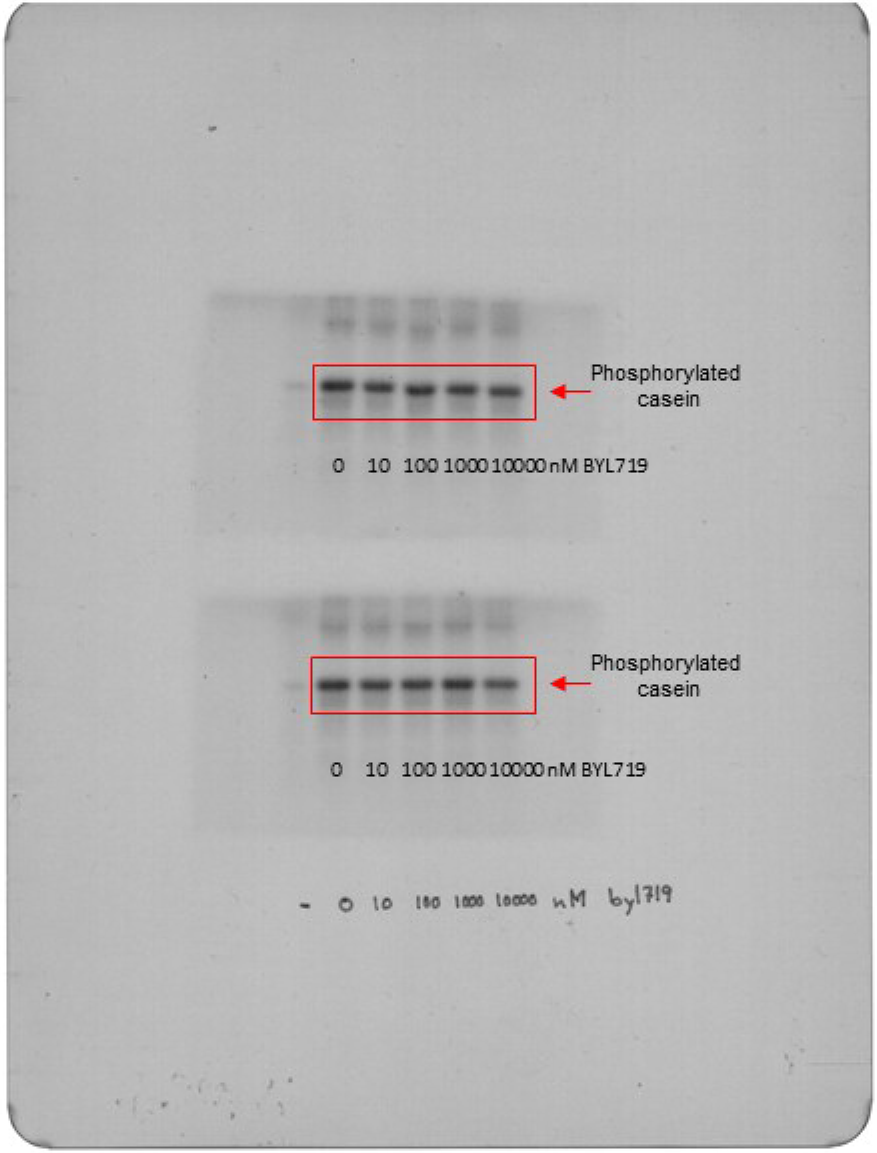

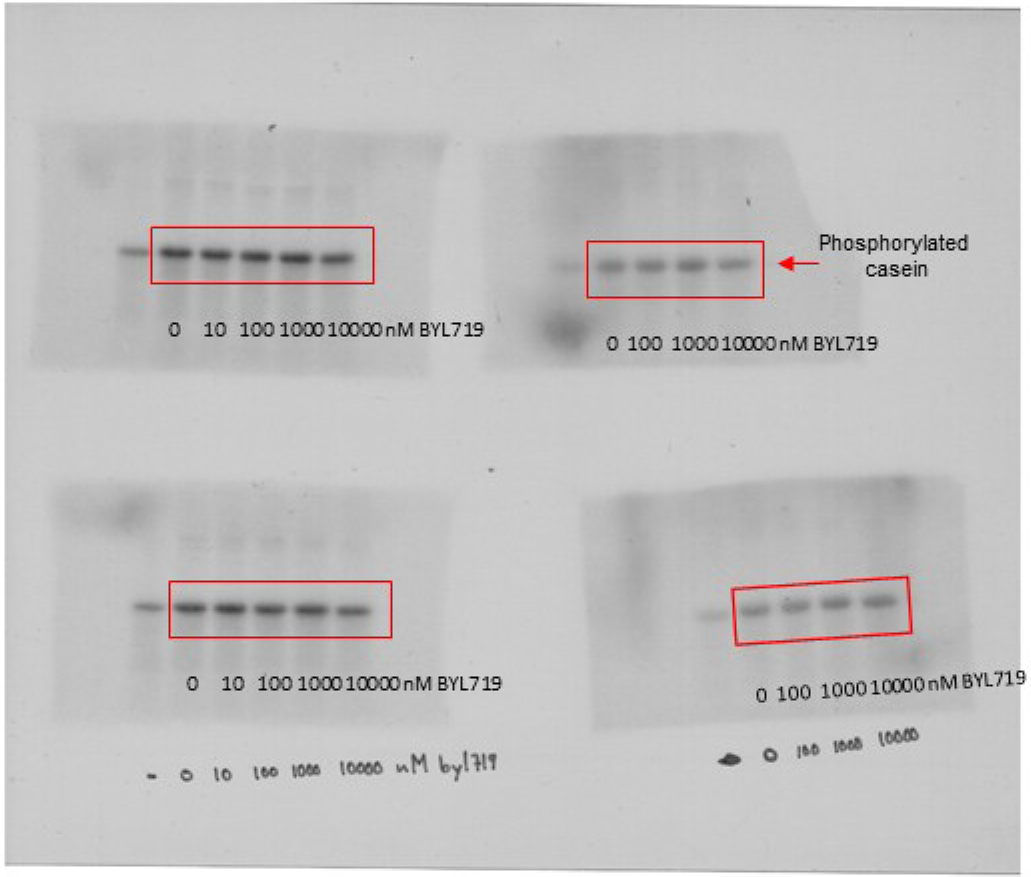

Figure 4D: Casein phosphorylation by ACVR1^{R206H} kinase. Phosphorylation was performed in the presence of ACVR1^{R206H} kinase and increasing concentrations of the PI3Kα inhibitor BYL719.
